# NLRP3 inflammasome activation is involved in manganese-induced immunotoxicity: Mn activates macrophage NLRP3 inflammasome

**DOI:** 10.3724/abbs.2021024

**Published:** 2021-12-23

**Authors:** Jing Ji, Haiyi Yu, Lei Liao, Jiaojiao Li, Kunming Qin, Jinyang Shen, Bo Guo, Xiaofu Shao, Jinming Ma, Jingquan Dong, Song Gao

**Affiliations:** Jiangsu Key Laboratory of Marine Biological Resources and Environment Jiangsu Key Laboratory of Marine Pharmaceutical Compound Screening Co-Innovation Center of Jiangsu Marine Bio-industry Technology School of Pharmacy Jiangsu Ocean University Lianyungang 222005 China

Manganese (Mn) is an essential trace element for the normal development of human body. It plays an important role in the regulation of cellular energy level, blood sugar balance, function of immunity, and bone growth, and works as an essential part of many indispensable enzyme functions. Meanwhile, overexposure to divalent manganese (Mn ^2+^) could cause a series of physiological and pathological dysfunctions, and Mn has been recognized as an environmental toxicant and an occupational hazard [1]. With the increasing exposure risk in environmental settings, such as contaminated food and polluted water and soil, Mn ^2+^-induced toxicities have become an important public health issue.

Neurotoxicity induced by Mn ^2+^ exposure has been extensively studied, and neuroinflammation has been recognized as the key player that significantly increases the risk of neurodegenerative disorders. Besides neurotoxicity, Mn ^2+^-induced immunotoxicity has also been observed. It has been shown that Mn ^2+^ exposure could enhance the activity of natural killer cells through type I interferons (IFNs) and elevate the humoral immune response in mice [2]. However, the underlying mechanism in Mn ^2+^-induced immunotoxicity is yet to be explored. As disruption of the immune system may impact the body defense against infectious diseases, oxidative stress, and tumorigenesis, etc. [3], Mn ^2+^-induced immunotoxicity can lead to a broad range of disorders and should be further investigated.

The innate immunity functions at the frontier of body responses to invading microbes or environmental stimuli. Macrophages are a chief player in the innate immunity that control inflammatory responses and secret important inflammatory cytokines [ 4, 5]. In macrophages, initiation of inflammatory responses involves the activation of inflammasomes. Inflammasomes are important for defending pathogens, but their inappropriate activation is harmful for potentially causing immune-related disorders. To understand the immunotoxicity of Mn ^2+^, study on inflammasomes of macrophages is a pivotal direction [ 6, 7].

In the present study, mouse peritoneal macrophages (PMs) were used as an *in vitro* model to investigate the immunotoxicity of Mn ^2+^. Female C57BL/6 mice (6–8 weeks old; Zhejiang Experimental Animal Center, Hangzhou, China) were individually housed in cages with a light/dark cycle of 12/12 h and free access to sterile food and water under the specific pathogen-free (SPF) condition of the Laboratory Animal Center of China Pharmaceutical University (Nanjing, China). All procedures have been approved by the Animal Care Commission of China Pharmaceutical University.

For collection and culture of resident PMs, PMs were harvested in cold PBS from the peritoneal cavities of the mice after intraperitoneally inoculated with 2.5 mL of 5% thioglycollate medium (Solarbio Science & Technology, Beijing, China) for 4 days. Then the PMs were suspended in RPMI 1640 medium containing 10% fetal bovine serum (FBS), 100 U/mL penicillin and 100 μg/mL streptomycin (Solarbio Science & Technology), and incubated in individual flat-bottom culture plates at 37°C in an incubator with 5% CO _2_. For western blot analysis, the cell culture supernatant was centrifuged at 1,500 *g* for 5 min to remove the cell debris. Subsequently, 1 mL of the supernatant was processed to obtain the protein sample using chloroform and methanol. The protein pellet was dissolved in 1% SDS. For the cell lysates, proteins were extracted using the RIPA Lysis Buffer containing 1 mM PMSF (Beyotime, Shanghai, China). The protein samples were subject to 15% SDS-PAGE and transferred to PVDF membranes. Membranes were blocked with 5% skimmed milk and incubated with indicated primary antibodies, followed by incubation with HRP-conjugated secondary antibodies (Proteintech, Wuhan, China). The signals were developed using the ECL Plus western blotting detection reagent (Merck Millipore, Bedford, USA). For RNA extraction and quantitative real-time PCR (qPCR), total RNA was isolated from PMs using the Total RNA Extraction Reagent (Vazyme, Nanjing, China). qPCR reactions were performed on a StepOne Real-Time PCR System (Thermo Fisher Scientific, Waltham, USA) using the qPCR SYBR Green Master Mix (Vazyme). *β-Actin* was used as the housekeeping gene control. The primer sequences are shown in Table 1. Data were presented as the mean±SEM of at least three independent experiments using Graphpad Prism 5.0. ANOVA Bonferroni multiple comparisons test, Tukey post-hoc test, and/or unpaired Student’s *t*-test were used with SPSS version 11.0. *P*<0.05 is considered as statistically significant.

**Table:** **Table1** Sequences of primers used in qPCR

Gene	GenBank acce-ssion No.	Primer sequence (5′→3′)
*IL-6*	NC_000071	Forward: TGCCTTCTTGGGACTGATGC
Reverse: GCAAGTGCATCATCGTTGTTC
*IL-12*	NM_001303244	Forward: GGAAGAGTCCCCCAAAAGCT
Reverse: CAGCAAAGGTGTCATGATGAACTT
*IL-18*	NM_008360	Forward: ACCAAGTTCTCTTCGTTGAC
Reverse: CTTCACAGAGAGGGTCACAG
*IL-1β*	NM_008361	Forward: AGGAGAACCAAGCAACGACA
Reverse: CTCTGCTTGTGAGGTGCTGA
*TNF-α*	NM_013693	Forward: GACGTGGAACTGGCAGAAGA
Reverse: GGCTACAGGCTTGTCACTCG
*NOD1*	NM_172729.3	Forward: GATTGGAGACGAAGGGGCAA
Reverse: CGTCTGGTTCACTCTCAGCA
*NOD2*	NM_145857.2	Forward: GCCAGTACGAGTGTGAGGAG
Reverse: GCGAGACTGAGTCAACACCA
*NLRP1*	NC_000077	Forward: ATAAACAAGCCACCCCCAGT
Reverse: TGTGCCCAATGTCGATCTCA
*NLRP3*	NM_145827.4	Forward: AGCCAGAGTGGAATGACACG
Reverse: CGTGTAGCGACTGTTGAGGT
*NLRC4*	NM_001033367	Forward: GCTCAGTCCTCAGAACCTGC
Reverse: ACCCAAGCTGTCAATCAGACC
*ACTIN*	NM_007393	Forward: GCCATGTACGTAGCCATCCA
Reverse: ACGCACGATTTCCCTCTCAG
*Caspase-1*	NC_000075.7	Forward: AACCACTCGTACACGTCTTGC
Reverse: ATCCTCCAGCAGCAACTTCA

**Figure 1:**
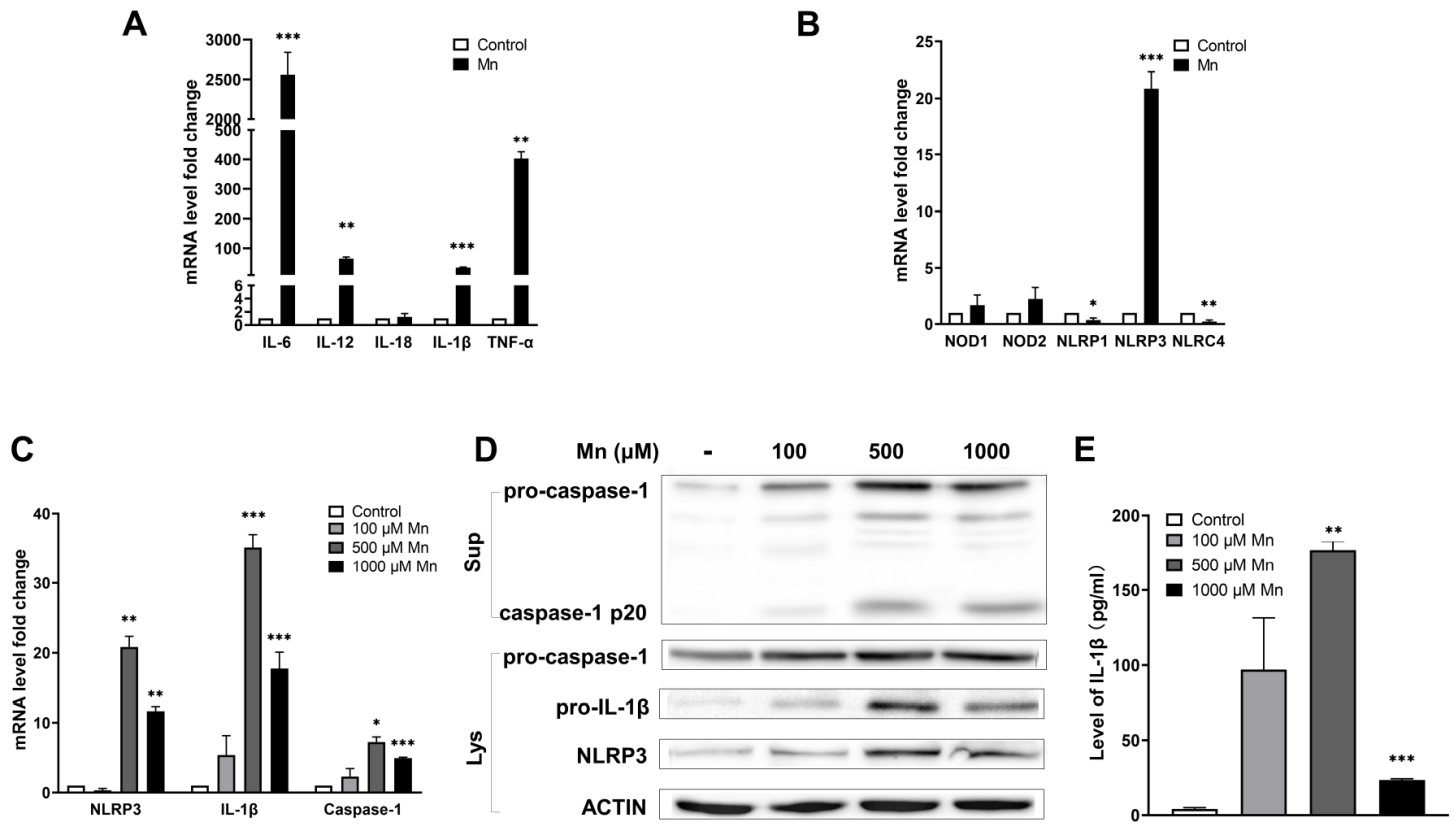
Figure1 **Activation of NLRP3 inflammasome in Mn ^2+^-induced PMs **For induction, PMs were treated with 500 μM of Mn2+or three different concentrations of Mn2+ for 12 h. (A) The relative transcription levels of IL-6, IL-12, IL-1β and TNF-α were measured by qPCR. (B) The relative transcription levels of NOD1, NOD2, NLRP1, NLRP3 and NLRC4 were measured by qPCR. (C) The relative transcription levels of NLRP3, IL-1β and caspase-1 were measured by qPCR. (D) Expressions of pro-caspase-1, cleaved caspase-1 (p20), pro-IL-1β and NLRP3 were analyzed by western blot analysis. Sup, supernatant. Lys, cell lysate. (E) Secretion of IL-1β to the supernatant was measured by ELISA. The data represented results from three independent experiments and were presented as the mean±SEM. *P<0.05, **P<0.01, ***P<0.001 compared with the negative controls that were not Mn2+-induced.

We found that in response to Mn ^2+^ exposure, the expression levels of IL-6, IL-12, IL-1β and TNF-α were significantly upregulated, while the expression of IL-18 remained unchanged ( Figure 1A). For the inflammasome-related genes, NLRP1 and NLRC4 were slightly down-regulated, and NLRP3 was greatly up-regulated ( Figure 1B). These results suggested that inflammasome response is involved in the Mn ^2+^ exposure-induced effects of PMs and NLRP3 inflammasome may play an important role in the Mn ^2+^-induced inflammation of PMs.

Mn ^2+^, at concentrations above 500 μM, greatly up-regulated three key components of NLRP3 inflammasome, including secretion of IL-1β, the activation of caspase-1 and the expression of NLRP3 in PMs ( Figure 1C). The protein levels of these key components were also increased, accompanied by the appearance of cleaved caspase-1 p20, with Mn ^2+^ induction as determined by western blot analysis ( Figure 1D). The release of active IL-1β to the supernatant was found to be induced by Mn ^2+^ exposure as revealed by ELISA ( Figure 1E). These results indicated that activation of NLRP3 inflammasome was part of the Mn ^2+^-induced inflammation in PMs.

**Figure 2:**
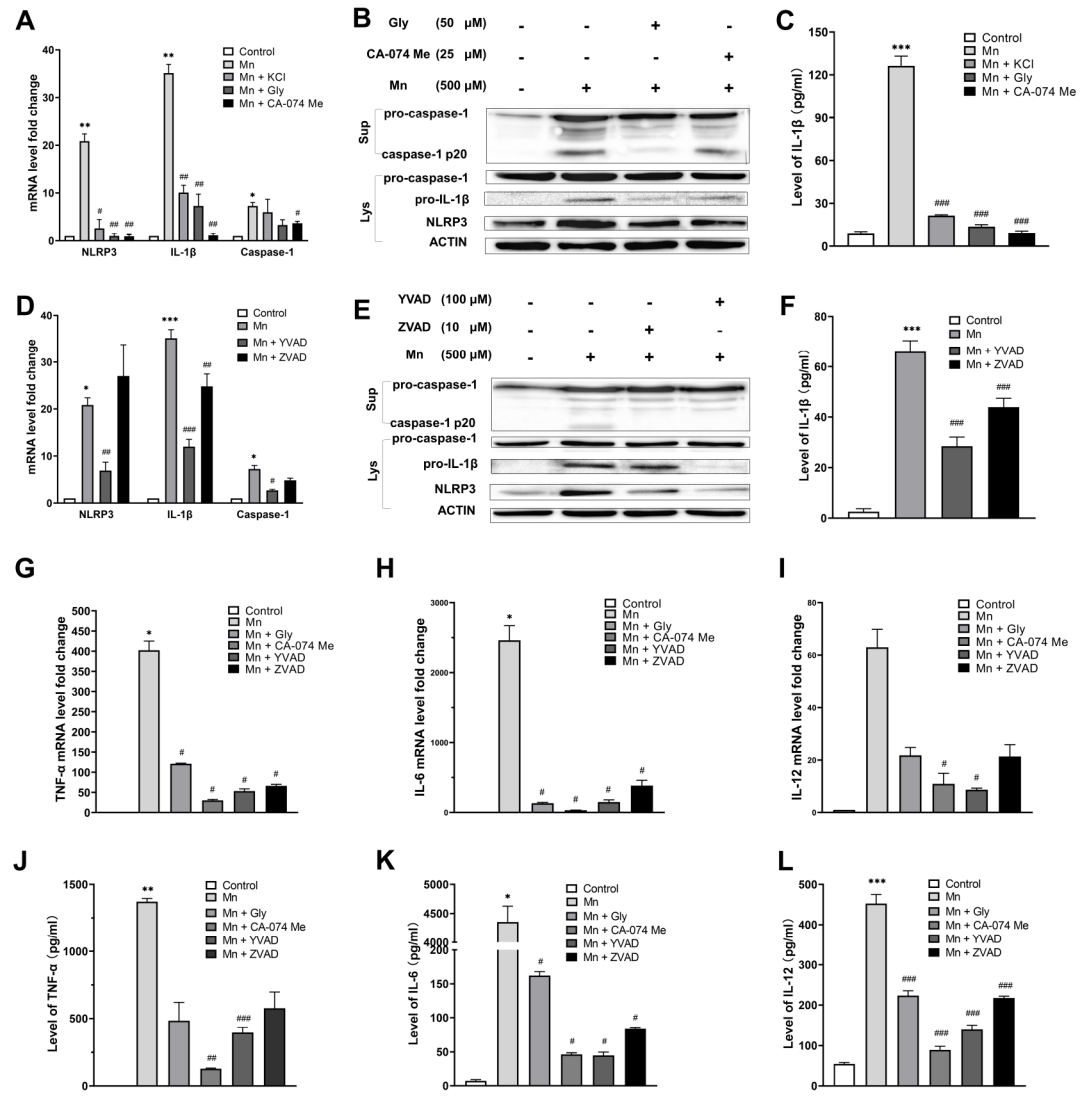
Figure2 **Inflammation in Mn ^2+^-induced PMs blocked by NLRP3, extracellular K ^+^, and caspase inhibitors **In samples with the inhibitors, PMs were treated with 50 μM of Gly, 25 μM of CA-074 Me, 100 μM of YVAD or 10 μM of ZVAD for 60 min, followed by induction with 500 μM of Mn2+ for 12 h. For treatment with extracellular K+, PMs were treated with 50 mM KCl for 60 min, followed by induction with 500 μM of Mn2+ for 12 h. (A,D) The relative transcription levels of NLRP3, IL-1β and caspase-1 were measured by qPCR. (B,E) Expression of pro-caspase-1, cleaved caspase-1 (p20), pro-IL-1β and NLRP3 were analyzed by western blot analysis. Sup, supernatant. Lys, cell lysate. (C,F) Secretion of IL-1β to the supernatant was measured by ELISA. (G–I) The relative transcription levels of TNF-α, IL-6 and IL-12 were measured by qPCR. (J–L) Secretion of inflammatory cytokines TNF-α, IL-6 and IL-12 to the supernatant was measured by ELISA. Data are presented as the mean±SEM from three independent experiments. *P<0.05, **P<0.01, ***P<0.001, compared with the negative controls that were not Mn2+-induced. #P<0.05, ##P<0.01, ###P<0.001, compared with the Mn2+-induced samples without inhibitor treatment.

We further confirmed the involvement of NLRP3 inflammasome in the Mn ^2+^-induced inflammation in PMs by using NLRP3 inhibitors. The elevated transcription levels of the three key components of NLRP3 inflammasome with Mn ^2+^ exposure were suppressed by NLRP3 inhibitors, i.e., Glibenclamide (Gly; inhibits K ^+^ efflux) and CA-074 methyl ester (CA-074 Me; inhibits cathepsin B) ( Figure 2A). The protein levels of NLRP3, IL-1β and cleaved caspase-1 p20 that were augmented with Mn induction were also decreased in the presence of the inhibitors ( Figure 2B). The secretion of IL-1β to the cell supernatant with Mn ^2+^ induction was also suppressed ( Figure 2C). These results indicated that NLRP3 inhibitors could restrain the expression of NLRP3, the activation of caspase-1, and the secretion of IL-1β, suggesting that NLRP3 plays an important role in the inflammation of Mn ^2+^-induced PMs.

Evidence have shown that K ^+^ efflux is a common signaling event of NLRP3 inflammasome activation, though there are exceptions [8]. To test if extracellular K ^+^ could suppress the inflammation, PMs were pre-treated with KCl before the Mn ^2+^ induction. Indeed, extracellular K ^+^ suppressed the inflammatory signals ( Figure 2A,C), suggesting the involvement of K ^+^ efflux in the Mn ^2+^-induced NLRP3 inflammasome activation.

Then we determined whether caspases play an essential role in the Mn ^2+^-induced inflammation by using Z-VAD-FMK (ZVAD; an inhibitor of pan-caspase) and Ac-YVAD-CHO (YVAD; an inhibitor of caspase-1 and caspase-4). The inhibitor YVAD had a more significant effect on the transcriptions of the three key components of NLRP3 inflammasome induced by Mn ^2+^ exposure than the inhibitor ZVAD ( Figure 2D). Western blot analysis and ELISA demonstrated the suppression of caspase-1 activation and IL-1β secretion by both caspase inhibitors, and YVAD had a more significant effect ( Figure 2E,F). These results suggest that caspases, particularly caspase-1, are required for the Mn ^2+^-induced inflammation in PMs.

Three additional inflammatory cytokines, including TNF-α ( Figure 2G,J), IL-6 ( Figure 2H,K) and IL-12 ( Figure 2I,L), showed elevated expression and secretion with Mn ^2+^ exposure in PMs, as confirmed by qPCR and ELISA. Moreover, their expression and secretion were significantly down-regulated by the NLRP3 and caspase inhibitors. Thus, the NLRP3 inflammasome signaling pathway plays a key role in the Mn ^2+^-induced inflammation in PMs by mediating the secretion of important inflammatory factors, including TNF-α, IL-6, IL-12, and IL-1β.

In summary, this study for the first time reported the activation of NLRP3 inflammasome in macrophages as a critical event of the Mn ^2+^-induced immunotoxicity. The NLRP3 inflammasome in PMs was activated by Mn ^2+^ exposure and mediated the activation of caspase-1 and the release of IL-1β. PMs rarely express pro-IL-1β without the presence of innate immunity inducers [9]. The expression of pro-IL-1β and secretion of its active form were significantly upregulated in PMs with Mn ^2+^ induction, and the relationship with activation of NLRP3 inflammasome was clearly demonstrated. The K ^+^ efflux was an upstream signal of the NLRP3 inflammasome activation. These observations are similar to the phenomenon in microglia of Mn ^2+^ exposure, in which the NF-κB pathway plays a critical role in the formation of NLRP3 inflammasome, and consequently causes the release of mature IL-1β and other inflammatory factors [10]. This suggests the involvement of NLRP3 inflammasome in the overall effect of Mn ^2+^-related toxicities. The NOD-like receptor protein 3-caspase 1 (NLRP3-CASP1) pathway could be a common therapeutical target in both immune and nervous systems for inflammatory damages caused by Mn ^2+^ exposure. Potential effective drug targets in this pathway should be screened in the future.
